# Neurorobotics for automotive manufacturing industry in era of embodied intelligence: a mini review

**DOI:** 10.3389/fnbot.2026.1796043

**Published:** 2026-03-03

**Authors:** Bangcheng Zhang, Qi Xia

**Affiliations:** 1 School of Mechatronic Engineering, Changchun University of Technology, Changchun, China; 2 Faculty of Mechanical and Electrical Engineering, Changchun Institute of Technology, Changchun, China

**Keywords:** automotive manufacturing, embodied intelligence, industrial robot, industry 5.0, neurorobotics

## Abstract

As automotive manufacturing advances toward the industrial 5.0 era, traditional rigid automation production models are transitioning toward the embodied intelligence paradigm. Confronted with mass customization, diverse products, and small-batch production, the environment of automotive manufacturing exhibits high dynamism and unstructured characteristics. Different from traditional industrial intelligence based on static, hard-coded logic, robots enhance their cognitive abilities through closed-loop interaction with dynamic environments, inspired by bionic neural mechanisms, this shift enables robots to perform flexible and reliable operations in complex production scenarios. This paper analyzes the core role and key technologies of neural intelligence algorithms in reshaping perception, decision, and execution of industrial robot, while providing a systematic review of industrial robot evolution within the automotive industry, and provides a reliable path for future development.

## 1 Introduction

The automotive industry is a representative of the manufacturing industry, as the demand has shifted to customization, and the production system faces severe challenges of multi-variety, small batch, and personalized customization. Under this background, the industrial robot, as the core execution unit in manufacturing, has undergone a profound transformation from a single repetitive task to multi-task collaboration, automation, and intelligence since it first moved from the laboratory to the production line in 1961, driven by rising labor costs and safety factors. Nowadays, modern automobile assembly lines usually deploy thousands of industrial robots, aiming at multi-dimensional optimization of scale, reliability, and cost.

However, although robot technology has made great progress, the traditional robot system still faces many bottlenecks in the process of stepping into the era of embodied intelligence. For now, the automotive production systems always rely on static environment assumptions, exhibiting an ability of insufficient generalization, especially in autonomous decision making when handling unstructured dynamic disturbances (Xie et al., 2024), such as mixed-model production lines, frequent process changes, and material shortages (Gao et al., 2022). Traditional control strategies achieved millimeter level precision (Wang et al., 2023), and it is a struggle to keep the millisecond- and second-level of real time dynamic responses, required by the actual production system, and exhibits a poor robustness (Zhang et al., 2025). Furthermore, the traditional frame-driven vision systems often suffer from issues of latency, consuming nearly half the total energy of a robot under the dual carbon goals.

**Figure 1:**
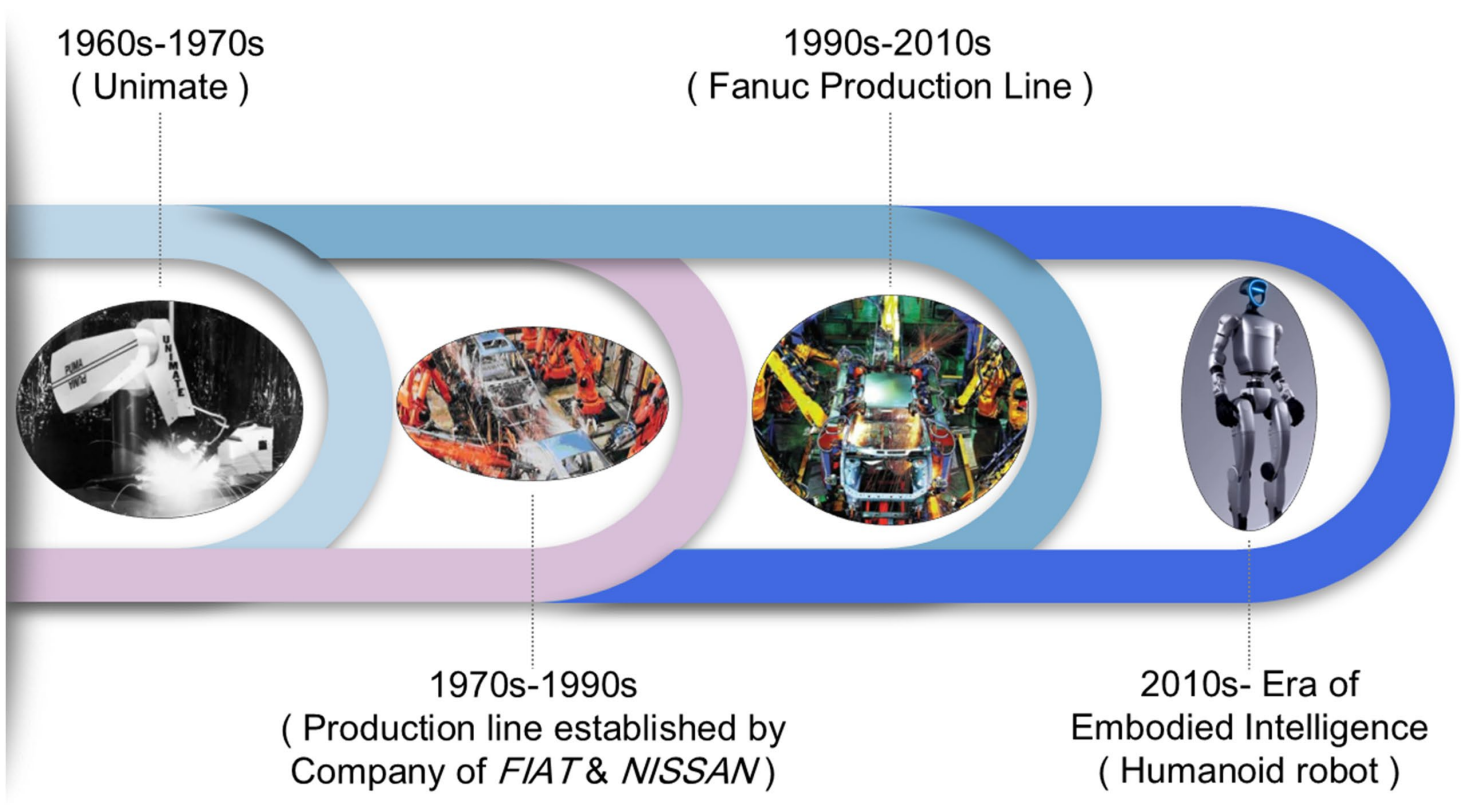
Evolution of industrial robots in automotive manufacturing.

Nevertheless, breakthroughs in neurorobotics offer new ways to overcome these challenges. Traditional industrial intelligence is limited by environmental models and relies on static, hard-coded logic, whereas embodied intelligence achieves smart perception, decision-making, and execution in robots through dynamic interaction with environment, combined with nonlinear modeling in neuro-robotics and feature learning driven by data (Andrea and Alessandro, 2025; Hu et al., 2025). In recent years, the framework of deep learning has been widely applied to path planning (Sun et al., 2024), visual inspection, and production process optimization (Liu et al., 2025). In contrast, while traditional optimization techniques face limitations when processing massive heterogeneous data, neuromorphic sensing systems (Zhao et al., 2022) and the control strategies incorporating physical information embedding (Eisa et al., 2025) demonstrate great potential, which achieved reducing energy consumption and enhancing system robustness. Figure 1 shows the evolution of industrial robots in automotive manufacturing.

This study conducted a systematic search of academic literature published between January 2021 and December 2025. We searched major academic databases such as IEEE Xplore, ScienceDirect, ACM Digital Library, SpringerLink, arXiv, and PubMed. The search topics encompassed neural model and automotive manufacturing. A total of 253 relevant records were obtained in the initial search. Through progressive screening of titles and abstracts, after removing duplicates and studies unrelated to automotive manufacturing scenarios, 29 articles were selected as the analysis objects of this mini review. Current literature lacks research on automotive manufacturing scenarios from the perspective of embodied intelligence, while focusing on how neuro-mechanistic approaches reconstruct the perception, decision, and action process of robots. The following chapters are organized as follows.

We review neuromorphic sensing and event-driven environment modeling techniques, discuss how to achieve efficient spatial and semantic representation by addressing pixel redundancy in Section 2. Section 3 analyzes neural control and planning with high dynamic adaptability, covering biologically inspired reinforcement learning, neurodynamic models, and adaptive strategies for physical information embedding. Section 4 focuses on the human-machine integration under green low-carbon manufacturing and industry 5.0 and discusses the research trends of energy efficiency optimization algorithms, model lightweight technology, and mutual cognitive human-machine collaboration architecture. Section 5 summarizes the challenges faced by the current research and looks forward to the future evolution direction of embodied intelligence in the field of automobile manufacturing. Section 6 concludes this study.

## 2 Neuromorphic sensing and event-driven environmental modeling

For automobile manufacturing of flexible flow line and frequent process changes, traditional environmental modeling based on geometric models, point cloud processing, or predefined states are inadequate. Leveraging knowledge graphs and graph neural networks (GNNs) to achieve semantic representation of the environment has become a research hotspot (Zhuang et al., 2023; Karthikeyan and Subashini, 2021). Systems infer latent causal relationships between equipment by modeling workshop states as graph structures, and then generating global decisions. Concurrently, digital twin technology integrating real-time data from physical entities into high-fidelity simulation environments, enables real-time monitoring (Song, 2023; Fatmah et al., 2025). There are some limitations in generalization of unstructured features, prompting exploration in few-shot learning, domain adaptation, and unsupervised frameworks. Nevertheless, nature of black-box possessed by neural networks conflicts with reliability demands of automotive manufacturing. Physics-informed neural networks (PINNs) embed physical laws as constraints within loss functions, exhibited a superior robustness in closed loop mechanical systems compared to traditional models (Eisa et al., 2025). Visual inspection always performed redundant computations on every pixel in each frame in traditional painting, in which over 95% of pixels remain static between frames. This rate-encoding typically resulted in high energy consumption and latency. Addressing this issue, researchers proposed a faster R-CNN-based visual system, which demonstrated giant efficiency advantages in automotive body painting defect detection (Rawat et al., 2021). However, there is some controversy about its scalability in complex automotive manufacturing scenarios. For instance, while the proposed model reduces pixel redundancy effectively, certain training methods face severe vanishing gradient issues. This leads to a decline in accuracy when handling long sequence tasks with high semantic complexity. Perception facilitates the evolution from image acquisition to interaction between the subject and environment, it will accelerate the transition from full computation to sparse perception.

## 3 Dynamic adaptive neural control and planning

Automotive production environments possess strong dynamics like emergency order insertions, equipment failures, and process changes, while traditional physics-based strategies struggle to balance dynamic responsiveness and system robustness, when confronted with random disturbances (Julian et al., 2024). In the issue of scheduling problems, traditional heuristic algorithms easily get stuck in local optima caused by high-dimensional state space, while reinforcement learning algorithms based on Q-learning showed a superior performance (Waseem and Chang, 2023). Moreover, for task planning of collaborative robots, a deep reinforcement learning (DRL)-based framework was proposed, incorporating multi-attribute hierarchical task networks and a revival mechanism, achieving task completion time reduction in complex assembly scenarios (Hou et al., 2024). However, the randomness of the framework in decision-making process still conflicts with the certainty requirement for actual automotive production. Industrial robots shall possess brain-like decision flexibility, rather than relying on pre-programmed rigid body dynamics. Considering issue of speed enhancement in data communication, Petrut et al. (2021) conducted a strategic development of the brainstem model, a model of the cerebellum was simulated on brain-inspired hardware, the verification was conducted through the SpiNNaker brain-like system and passed the benchmark verification of NEST software, results demonstrated that the model reduced the peak communication load by 41%. Furthermore, addressing nonlinear dynamic control challenges in assembly tasks, a fuzzy PID system integrated with spider monkey optimization (SMO) was proposed, achieving precise end effector control (Alka et al., 2021). There are some successful cases for achieving embodied intelligence of robots, such as acquired skills through observation or demonstration (Park et al., 2023). Zhao et al. (2022) developed a brain-inspired hierarchical system for online gesture recognition and action learning, named spiking gating flow (SGF). By integrating feature extraction and event-driven training, it showed exceptionally high spatio-temporal feature utilization efficiency. However, the end-to-end models rely heavily on high-fidelity simulations, even slight modeling deviations caused by physical parameter influences often show a bottleneck when transferring from virtual environment to real physical space, due to a policy performance variation. Currently, unstructured disturbances in real scenarios are complex, the neural control strategies still require in-depth exploration for multi-objective trade-offs and transfer implementation when addressing this problem.

## 4 Manufacturing of automotive industry in industry 5.0

Driven by industry 5.0 and carbon neutrality, the goals of synergy, sustainability, and human centricity are of equal importance. Energy reduction generally relies on hardware improvements, offline planning based on rigid body dynamics, which neglects the nonlinear interactive effects in dynamic environments. In contrast, the neural intelligence algorithm-based trajectory energy consumption models achieved over 30% energy consumption reduction according to Yao et al. (2024), it was integrated into industrial internet of things (IIoT) platforms and digital twin systems to accomplish full lifecycle energy consumption management (Chandrasekaran and Rajesh, 2024). Wu and Corves (2025) proposed an adaptive optimal trajectory tracking control framework, achieved a dynamic equilibrium of control performance and energy consumption while maintained operational precision. Addressing computational energy consumption when deploying deep learning models at the edge, Arijit et al. (2024) applied a structured large-scale lottery hypothesis (LTH) to remove sparse layers from neural networks, achieved overall system energy loss reduction.

Industry 5.0 emphasizes deep integration between human and robots (Zheng et al., 2022), nevertheless, due to the inherent uncertainty in human operations, establishing efficient mutual cognition mechanism remains a challenge. To solve unpredictable human interactions, such as turn-taking prediction in human-robot collaborative assembly, Xu et al. (2023) proposed a spiking neural network (SNN) based on the Izhi neuron model, the proposed spatio-temporal prediction and planning framework (STAP-PPF) enhanced coordination through dynamically updated paths. Although this method showed certain advantages when handling standard collaborative actions, when confronted with worker fatigue, variations in worker skill levels, and other non-standard disruptive movements, the predictive accuracy will decline exponentially. For achieving deep reasoning for complex collaboration, a vision reasoning-based mutual cognition HRC approach was proposed (Jared and Gloria, 2023), which constructed domain-specific knowledge graphs and leveraged graph embedding techniques, results showed a good performance. The introduction of knowledge graph and semantic reasoning enhances the depth of understanding, but the resulting massive data significantly increases time delays, and adding production risk. There is still a certain gap between the simulation in the laboratory and the actual industrial deployment, embodied intelligence must be integrated into mature industrial internet architectures to meet the real-time and reliability demands of industry 5.0. For instance, in practical engineering applications within automotive manufacturing, He et al. (2024) proposed a long short-term memory neural network (LSTM) for the state recognition of the industrial robot, this model was deployed in a real-time production environment for continuous evaluation for state recognition of industrial robots. This application delivers data support for enhancing production process transparency. By identifying abnormal conditions during manufacturing, it strengthens resilience within systems, achieves full traceability across the entire process under industry 5.0. Robots are evolving from production tools to cognitive collaborators with deep semantic understanding capabilities. This transformation goes beyond improvements in technical metrics, but lies in how to achieve a deep integration of domain prior knowledge and data-driven models in the context of high-dimensional, heterogeneous, and massive data. A unified cross-modal cognitive framework that integrates multi-source heterogeneous information is not to break the perception barriers of machine collaboration, but the ability to realize the semantic understanding and flexible decision-making ability of embodied agents under complex production conditions. Table 1 shows a multi-dimensional summary of neurorobotic methodologies in automotive manufacturing (2021–2025).

**Table 1:** Summary of neurorobotic methodologies in automotive manufacturing (2021–2025).

Dimension	Mechanism	Ref.	Application	Key advantages
Perception and environment modeling	SNN/SGF	Zhao et al. (2022)	On-line action recognition	Reduce pixel redundancy and perceived latency.
	Faster R-CNN	Karthikeyan and Subashini (2021)	Mechanical fastener assembly	Recise position and detection of bolts in body-in-white assembly lines.
	CNN + Bi-LSTM	Zhuang et al. (2023)	Binocular camera pose estimation	Improve posture accuracy in complex working conditions.
	SNN	Fatmah et al. (2025)	Edge computing target tracking	Improve real-time detection performance at the edge.
	CNN-SLAM	Song (2023)	Autonomous navigation in unknown environments	Improve map construction and path planning capabilities in dynamic scenarios of robot.
	Faster R-CNN	Rawat et al. (2021)	Defect detection after painting	Improve the identification efficiency and detection accuracy of abnormal defect types.
	PINN	Eisa et al. (2025)	Closed-loop mechanical system identification	Improve model consistency and robustness.
	PID-SMO	Alka et al. (2021)	Robotic arm end position control	Effectively solve nonlinear and uncertain dynamic problems in control systems.
	Multi-algorithm fusion control strategy	Zhang et al. (2025)	Intelligent welding seam tracking system	Enhance adaptive ability during the welding process.
	Intelligent Smooth Sliding Mode Control	Gao et al. (2022)	Electrophoretic coating conveying control	Achieve synchronized flexibility in multi-robot trajectories.
	Q-Learning	Waseem and Chang (2023)	Flexible manufacturing system (FMS) scheduling	Independently learn optimal scheduling strategies to effectively cope with throughput fluctuations.
	Adaptive optimal trajectory tracking control	Wu and Corves (2025)	Delta Robot Control	Dynamically balance accuracy and energy consumption.
	YOLOv5 + human demonstration learning	Park et al. (2023)	Autonomous robot picking platform	Realize the rapid positioning and flexible grasping operation of the target object.
	Structured Lottery Ticket Hypothesis (LTH)	Arijit et al. (2024)	Model compression and energy saving	Reduce inference energy consumption while maintaining accuracy.
	LSTM + IIoT	He et al. (2024)	Robot status recognition and energy consumption management	Realize performance monitoring and production optimization decision support throughout the life cycle.
	SNN/Izhi	Xu et al. (2023)	HRC assembly rotation forecast	Predict human uncertainty in advance and improve the efficiency of human-computer collaboration and interaction.
	Spatio-Temporal Prediction and Planning Framework	Jared and Gloria (2023)	Collaborative robot active path planning	Predict the sequence of human body movements and generate a trajectory with optimal time and safety.
	Data augmentation-Gradient boosting decision tree	Wang et al. (2023)	Manufacturing quality prediction	Dynamically balance training time and prediction accuracy to support optimization decision-making.

## 5 Challenges and future directions

Even if there are some potentials in reconfiguring perception, decision-making, and execution in automotive manufacturing, transferring neurorobotics from laboratory to industrial scenarios faces challenges.

### 5.1 Challenges

Although neurally grounded intelligent algorithms provide theoretical support for industrial robots in practical applications, they still face profound challenges in the nonlinear, strongly coupled, and highly dynamic industrial environment of automotive manufacturing.

The automobile manufacturing requires traceability of the whole process, while the neural network relies on the black box feature in the production system, in key workstations, it is difficult to deconstruct the weighted decision-making schemes into explanations that align with actual production rules, therefore, this lack of transparency adding traceability analysis and troubleshooting of failure. At the same time, manufacturing systems have not established a comprehensive certification framework, thus, deploying end-to-end learning models in real-world production scenarios involving human-machine interaction still faces significant barriers.

Robot often fails to comprehend underlying environment intentions, the systemic capabilities akin to human experts, such as cognitive coordination and decision still need further exploration (Lian et al., 2024). It is a challenge for achieving truly safe and efficient human-robot deep collaboration without bidirectional mutual cognition mechanism.

Advanced architectures such as transformers or graph neural networks enhance the depth of semantic perception, however, massive parameter counts cause inference delays and the addition of response time. Besides, deploying such models on computationally constrained edge devices requires sacrificing inference accuracy or incurring costly investments. At the same time, research often overlooks the application costs of models, including data management, offline training, and energy consumption from hardware upgrades. For instance, cycle time savings by algorithm may be offset by maintenance costs when the consumption is not effectively optimized.

Existing deep learning models heavily rely on training data with specific distributions. Confronted with unstructured perturbations, such as new vehicle models, workshop setting changes, or deformations in flexible materials, the generalization error of the model increases exponentially. Moreover, the robust strategies trained in simulated environments cannot fully achieve good performance in real physical scenarios.

Industry 5.0 is not merely an iteration of technology but a reshaping of the workforce structure. Currently, there is a significant cognitive gap between engineers and AI experts. The self-learning capabilities of neural networks require skill in data processing and parameter tuning for the operator, which increases personnel training expenditures, moreover, integrating expert knowledge with black-box strategies to achieve collaborative operations remains a significant technical challenge.

### 5.2 Future directions

Automotive manufacturing requires high cycle time, low latency requirements, future research shall focus on the computational efficiency of biological neural systems and advanced cognitive frameworks. Brain-inspired control systems and dynamic compensation integrated with neural networks enable microsecond-level monitoring of anomalies, significantly enhancing system robustness.

To address the trust deficit in human-robot collaboration, it is necessary to enhance the cross-modal understanding and knowledge representation of robots. Developing large language models to extract deep semantic information, transforming non-standard gestures, voice commands, and ambiguous operational intentions of workers into structured task graphs, thus achieving the improvement of collaborative efficiency. Simultaneously, neural networks are employed to fuse the acquired high-dimensional features with digitized expert knowledge, thereby enhancing the logical reasoning capabilities and improving the interpretability of decisions of the system.

Mixed-line production of multiple vehicles increases downtime for training, the exploration of industrial foundation models (IFMs) shows advantages in dealing with this issue. Through continuous learning and experience accumulation across workstations, physical laws and expert knowledge are integrated into neural networks, the framework integrated structured knowledge shortens deployment cycles for new tasks with generalization capabilities.

## 6 Conclusion

This mini review systematically argued the industrial robots in automotive manufacturing, different from the previous review that focused on the accuracy of algorithms, we outlined how robots in neurally grounded intelligent algorithms affecting the performance of the production system. Although the path to fully autonomous, self-evolving faces numerous challenges, such as computational bottlenecks and safety certification, with the integration of brain-inspired architectures and development of industrial large language models, future robots shall achieve a truly human-centered, green, and sustainable intelligent manufacturing system in the era of industry 5.0.
