# Correction: Neurorobotics for automotive manufacturing industry in era of embodied intelligence: a mini review

**DOI:** 10.3389/fnbot.2026.1829525

**Published:** 2026-04-02

**Authors:** Bangcheng Zhang, Qi Xia

**Affiliations:** 1 School of Mechatronic Engineering, Changchun University of Technology, Changchun, China; 2 Faculty of Mechanical and Electrical Engineering, Changchun Institute of Technology, Changchun, China

**Keywords:** automotive manufacturing, embodied intelligence, industrial robot, industry 5.0, neurorobotics

In the published article, affiliation 1 and 2 were erroneously ordered. The correct order of affiliations should have been: “1. School of Mechatronic Engineering, Changchun University of Technology, Changchun, China, 2. Faculty of Mechanical and Electrical Engineering, Changchun Institute of Technology, Changchun, China.”

In the published article, in the Funding statement, the funding source “Major Science and Technology Special Project of Jilin Province and Changchun City, (Grant No. 20250306008ZD) to BZ” was erroneously omitted. The corrected Funding statement appears below.

## Funding

The author(s) declared that financial support was received for this work and/or its publication. The research was funded in part by Major Science and Technology Special Project of Jilin Province and Changchun City, (Grant No. 20250306008ZD) to BZ.

The original article has been updated.

